# Therapeutic outcome of active management in male patients with detrusor underactivity based on clinical diagnosis and videourodynamic classification

**DOI:** 10.1038/s41598-021-04237-0

**Published:** 2022-01-10

**Authors:** Cheng-Ling Lee, Jia-Fong Jhang, Han-Chen Ho, Yuan-Hong Jiang, Yuan-Hsiang Hsu, Hann-Chorng Kuo

**Affiliations:** 1 Department of Urology, Hualien Tzu Chi Hospital, Buddhist Tzu Chi Medical Foundation, 707, Section 3, Chung-Yang Road, Hualien, Taiwan; 2 Department of Anatomy, Tzu Chi University, Hualien, Taiwan; 3 Department of Pathology, Hualien Tzu Chi Hospital, Buddhist Tzu Chi Medical Foundation, Hualien, Taiwan; 4 Tzu Chi University, Hualien, Taiwan

**Keywords:** Health care, Urology

## Abstract

Detrusor underactivity (DU) could be resulted from many different etiologies. Patients with DU might have reduced bladder sensation, low detrusor contractility, and large post-void residual volume. This study analyzed therapeutic outcome of active management for male DU patients, based on clinical and urodynamic characteristics. Male DU patients aged > 18 years old were retrospectively reviewed from the videourodynamic study (VUDS) records in recent 10 years. The patients’ demographics, VUDS results, treatment modalities, and treatment outcome were analyzed. The treatment outcomes were compared among patients with different DU subgroups, clinical diagnosis and treatment modalities. Patients with voiding efficiency of > 66.7% were considered having a successful treatment outcome. For comparison, 30 men with normal VUDS finding served as the control arm. Most of the DU patients had reduced bladder sensation. The reduced bladder sensation is closely associated with low detrusor contractility. After active treatment, a successful outcome was achieved in 68.4% of patients after bladder outlet surgery, 59.1% after urethral botulinum toxin A injection, and 57.6% after medical treatment, but only 18.2% after conservative treatment. A successful treatment outcome was achieved in patients with an intact detrusor contractility, either low (69.2%) or normal voiding pressure (81.8%), and in patients with a normal or increased bladder sensation (78.1%). However, patients with detrusor acontractile (41.3%) or absent bladder sensation (17.9%) had less favorable treatment outcome after any kind of urological management. This study revealed that active management can effectively improve voiding efficiency in patients with DU. The normal bladder sensation, presence of adequate detrusor contractility, and bladder outlet narrowing during VUDS provide effective treatment strategy for DU patients. Among all management, BOO surgery provides the best treatment outcome.

## Introduction

Detrusor underactivity (DU) is a urodynamic term that describes a group of patients who have difficulty in initiating, sustaining, and completing urination with low detrusor contractility and the aid of abdominal pressure. According to the International Continence Society definition, DU is a condition with bladder contraction of reduced strength and/or duration, resulting in prolonged bladder emptying and/or failure to achieve complete bladder emptying within a reasonable time^1^. There are multiple etiologies for DU, including neurogenic, myogenic, bladder outlet obstruction (BOO), ageing, and many systemic medical diseases^2^. Clinically, patients with DU may present with urinary retention, difficult initiation, slow stream and straining to urinate, or incomplete emptying^2^. Urodynamic study of DU usually reveals a non-contractile detrusor, a low pressure, or poorly sustained detrusor contraction associated with a poor flow rate, with or without a large post-void residual (PVR) volume^3^.

Patients with urodynamic DU might also have reduced bladder sensation causing incomplete voiding because they lack a fullness sensation^4^. Some DU patients have increased bladder sensation or detrusor overactivity (DO), which causes voiding at small bladder capacity resulting in low contractility. Age has been thought to be one obvious and important factor related to DU^5^. In addition, patients with chronic central nervous system (CNS) disorders, such as stroke or Parkinson’s disease, might have DU due to their lack of a bladder fullness sensation or other facilitation of urination^6^. Patients with chronic bladder outlet obstruction (BOO) could also develop DU and large PVR. The underlying pathophysiology for each DU subgroup might be different and attributable to different detrusor contractility and bladder outlet resistance^7,8^.

Urination is a complex process starting from relief of CNS inhibition, through an intact spinobulbospinal tract in the spinal cord and peripheral nerve, to adequate opening of the bladder neck, a urethral smooth and striated sphincter, relaxation of the pelvic floor muscles, a patent anterior urethra, and sustained detrusor contractility. Any abnormality in the neuromuscular complex of the urination control will lead to DU. Therefore, the pathophysiology of DU may involve pre-vesicle, vesicle, and post-vesicle factors^9–11^. However, although we might realize the possible pathophysiology of DU in patients with urinary retention, management of DU is a challenge to urologists.

Previous research on DU has usually focused on voiding detrusor pressure (Pdet) and voiding efficiency (VE) by conducting pressure–flow studies. However, the perception of bladder filling and CNS facilitation might be as important as detrusor contractility^12–14^. A videourodynamic study (VUDS) previously showed that bladder sensation during the storage phase, detrusor contractility, and bladder outlet appearance during the voiding phase could be clearly observed^3^. The results of VUDS in DU may provide insight into the pathophysiology of lower urinary tract dysfunction, and guide the therapeutic strategy to voiding function recovery. The study aim was to investigate the VUDS characteristics of male patients with DU and assess the treatment outcome based on different active managements and urodynamic findings, especially on the bladder sensation, detrusor contractility and VUDS characteristics.

## Methods

### Patient enrolment

A total of 298 male patients aged > 18 years old who could not spontaneously void (excessive urinary retention), strained to void with a low maximum flow rate (Qmax), or had a large PVR (> 33% of total bladder capacity) from the VUDS patient records over the most recent 10 years were retrospectively reviewed. Patients with spinal cord lesions, such as spinal cord injury, multiple sclerosis, or myelopathy, were not included. The patients’ demographics, VUDS results, treatment modalities [such as oral medication, indwelling catheter, clean intermittent catheterization (CIC), urethral sphincter botulinum toxin A (BoNT-A) injection], surgical treatments [such as transurethral incision of the bladder neck (TUI-BN), transurethral incision or resection of the prostate (TUIP or TURP), optic urethrotomy], and treatment outcome were analyzed. The DU patients were classified as subgroups with detrusor acontractility (DA, n = 147), low detrusor contractility (LDC, n = 125), and normal detrusor contractility (NDC, n = 26). For comparison, 30 men with VUDS-proven normal lower urinary tract function served as the control arm. The urodynamic parameters in different DU subgroups and different clinical disorders were compared. The treatment outcomes were also compared between patients in different DU subgroups. This study was approved by the Research Ethics Committee of Hualien Tzu Chi Hospital, Buddhist Tzu Chi Medical Foundation (IRB: 103-145-A). Informed consent was waived by the Research Ethics Committee of Hualien Tzu Chi Hospital, Buddhist Tzu Chi Medical Foundation due to the retrospective nature of the analysis. All methods were performed in accordance with the relevant guidelines and regulations.

### Videourodynamic classification of DU

VUDS was performed by using a standard procedure according to the guidelines of good urodynamic practice recommended by the International Continence Society^1^. The first sensation of filling (FSF), full sensation (FS), urge sensation (US), cystometric bladder capacity (CBC), bladder compliance, voiding Pdet, Qmax, voided volume (Vol), and PVR volume were recorded. Images of the bladder and bladder outlet during voiding, including the bladder neck opening or narrowing, opening or narrowing prostatic urethra, poor relaxation of the external sphincter (PRES), and distal urethral narrowing, were reviewed. All DU patients should have a bladder contractility index (BCI, defined as Pdet + 5 × Qmax) of < 100^15^. The VE was calculated according to the equation: Vol/Vol + PVR.

Bladder sensation was presented as a percentage of the CBC. If the FSF/CBC was < 30%, increased bladder sensation (IBS) was defined. If patients had a FSF/CBC ratio of 30%–50%, the bladder sensation was considered to be normal. If the FSF/CBC was > 50%, reduced bladder sensation was considered. However, if FSF/CBC was 100%, the bladder sensation was defined as absent. For Pdet ≤ 5 cmH_2_O, the detrusor contractility was defined as DA; for Pdet ≥ 30 cmH_2_O, NDC was considered; for Pdet > 5 but < 30 cmH_2_O, LDC was considered. The classification of DU in this study is basically arbitrarily but is still based on clinically relevant criteria. Patients with a detrusor pressure of < 5 cmH_2_O indicated very low or absent contractility, patients with a Pdet ≥ 30 cmH_2_O but incomplete bladder emptying indicated having normal but not sustainable detrusor contractility. The patients with Pdet > 5 but < 30 cmH_2_O had residual but low contractility. The variables of bladder sensation, detrusor contractility, and bladder outlet conditions were grouped and these VUDS findings were composed to make a different VUDS diagnosis of DU.

After the VUDS, patients with DU were classified according to the pathophysiology of the CNS lesion (such as stroke, dementia, Parkinson’s disease, degenerative diseases), bladder sensation (absent, reduced, normal, IBS), detrusor contractility (DA, LDC, NDC), and the bladder outlet appearance during voiding in VUDS (no BOO, BN obstruction, benign prostatic obstruction, and PRES). Patients with DU could also have any of these abnormalities of urinary control. The DU patients were classified according to their different etiology and the VUDS characteristics, and the parameters and treatment outcomes of the different DU subgroups were compared.

### Management of voiding dysfunction in DU patients

The patients with urodynamically proven DU were first treated conservatively with an indwelling Foley catheter, CIC, or by medication with an alpha-blocker if there were no evidence of BOO to reduce bladder outlet resistance. If the initial conservative treatment failed, VUDS was routinely performed to investigate the lower urinary tract dysfunction underlying DU. TUI-BN was suggested if a tight BN was noted during VUDS regardless of if there was detrusor contractility. If there was evidence of prostatic urethral narrowing, the patients were suggested to undergo TUI-P or TURP if their prostate volume was < 30 ml or ≥ 30 ml, respectively. When the bladder neck and prostatic urethra were open during voiding (spontaneously or by abdominal straining) but a tight urethral sphincter was noted, PRES was considered, and a urethral sphincter BoNT-A injection at a dose of 100 U was recommended. Patients with DU and anterior urethral stricture were treated with urethral dilatation or optic urethrotomy. If a patient did not agree to undergo bladder outlet procedures, the patient was treated conservatively by CIC or indwelling Foley catheter and follow-up at a urological clinic.

### Treatment outcome assessment

Treatment outcomes were assessed by chart review in the proceeding 1 year and graded from 0 to 3. Patients who needed an indwelling Foley catheter, continuing CIC, or had a VE < 33% were considered to have not responded adequately to treatment (grade 0). Patients who were able to urinate (spontaneously or by abdominal straining) with a VE 33–66.7% were considered to be mildly improved (grade 1). Patients who could urinate with a VE > 66.7% but < 90% were considered moderately improved (grade 2). Those who could urinate spontaneously without abdominal straining and having a VE > 90% were considered to have markedly improved (grade 3).

Continuous variables were expressed as the mean and standard deviation, and categorical data were presented as the number and percentage of patients. The chi-square test for categorical variables and the Wilcoxon rank-sum test for continuous variables were used to determine *p* values for statistical comparisons between groups. Post-hoc analysis of variables among different subgroups was also performed. We also selected 30 male patients each with normal VUDS findings as controls for comparison. All statistical assessments were two-sided and considered to be significant at *p* < 0.05. All calculations were performed by using SPSS for Windows (SPSS for Windows, Version 16.0.; SPSS Inc., Chicago, IL).

### IRB

This study was approved by the Research Ethics Committee of Hualien Tzu Chi Hospital, Buddhist Tzu Chi Medical Foundation (IRB: 103-145-A). Informed consent was waived by the Research Ethics Committee of Hualien Tzu Chi Hospital, Buddhist Tzu Chi Medical Foundation because of the retrospective study design.

## Results

A total of 298 male patients and 30 controls were analyzed. The distributions of DU by detrusor contractility and VUDS findings and urodynamic parameters are presented in Table 1. Compared with those in the controls (46.1 ± 15.4%), bladder sensations by ratio of FSF/CBC were significantly greater in patients with DA (63.2 ± 23.3%, p < 0.001), LDC (55.5 ± 18.4%), and NDC (57.70 ± 19.5%); whereas the Pdet was significantly lower in patients with DA (1.31 ± 2.07 cmH_2_O) and LDC (15.0 ± 6.34 cmH_2_O), but not NDC (33.9 ± 3.98 cmH_2_O). In patients with DU, the Pdet was positively correlated with Qmax (*r* = 0.539, *p* = 0.000) and negatively correlated with PVR (*r* =  − 0.397, *p* = 0.000), FSF (*r* =  − 0.233, *p* = 0.000) and FS (*r* =  − 0.148, *p* = 0.006). The BCI and VE were all significantly lower in patients with DA (6.98 ± 14.3, 9.79 ± 23.1), LDC (31.3 ± 20.1, 27.2 ± 32.2), and NDC (52.7 ± 15.9, 30.8 ± 28.5) compared with the controls (133.1 ± 22.8, 96.7 ± 4.56), all with significantly elevated PVR.

**Table 1:** Urodynamic parameters in different subgroups of detrusor underactivity in male patients and normal controls.

	DA (n = 147)	LDC (n = 125)	NDC (n = 26)	Controls (n = 30)	p value
Age	70.8 ± 13.7	70.9 ± 13.3	73.8 ± 12.7	59.9 ± 5.9	0.000
FSF/CBC %	63.2 ± 23.3	55.5 ± 18.4	57.70 ± 19.5	46.1 ± 15.4	0.000
FS/CBC %	87.8 ± 16.2	84.9 ± 15.2	86.3 ± 15.1	75.7 ± 13.5	0.002
US/CBC %	99.1 ± 5.0	98.8 ± 4.7	98.6 ± 5.0	94.5 ± 12.1	0.002
CBC (ml)	413 ± 192	360 ± 182	328 ± 138	502 ± 108	0.000
PVR/CBC %	115 ± 80.9	91.7 ± 97.5	90.7 ± 65.1	4.0 ± 5.5	0.000
Pves (cmH_2_O)	31.1 ± 35.9	35.1 ± 27.8	43.2 ± 16.6	50.4 ± 30.5	0.014
Pdet (cmH_2_O)	1.31 ± 2.07	15.0 ± 6.34	33.9 ± 3.98	32.6 ± 12.6	0.000
Qmax (ml/s)	1.13 ± 2.78	3.26 ± 3.71	3.77 ± 3.15	20.1 ± 3.1	0.000
Volume (ml)	32.3 ± 85.5	82.6 ± 107	98.5 ± 115	485 ± 103	0.000
PVR(ml)	378 ± 205	278 ± 204	229 ± 150	17.3 ± 24.8	0.000
Compliance	70.9 ± 105	62.7 ± 78.7	77.9 ± 115	68.2 ± 57.3	0.842
BCI	6.98 ± 14.3	31.3 ± 20.1	52.7 ± 15.9	133 ± 22.8	0.000
VE (%)	9.79 ± 23.1	27.2 ± 32.2	30.8 ± 28.5	96.7 ± 4.56	0.000
DO	8 (5.4%)	43 (34.4%)	12 (46.2%)	4 (13.3%)	0.000
Tight BN	69 (46.9%)	40 (32.0%)	8 (30.8%)	0 (0%)	0.000
Tight PU	19 (12.9%)	13 (10.4%)	4 (15.4%)	0 (0%)	0.124
Tight ES	48 (32.7%)	32 (25.6%)	5 (19.2%)	0 (0%)	0.002
ISD	10 (6.8%)	9 (7.2%)	0 (0%)	0 (0%)	0.328

*DA* detrusor acontractile, *LDC* low detrusor contractility, *NDC* normal detrusor contractility, *FSF* first sensation of filling, *CBC* cystometric bladder capacity, *FS* full sensation, *US* urge sensation, *PVR* post-void residual, *Pves* intravesical pressure, *Pdet* detrusor pressure, *Qmax* maximum flow rate, *BCI* bladder contractility index, *VE* voiding efficiency, *DO* detrusor overactivity, *BN* bladder neck, *PU* prostatic urethra, *ES* external sphincter, *ISD* intrinsic sphincter deficiency.

The distributions of DU patients with different clinical diseases and the relationship with reduced bladder sensation and bladder outlet conditions, such as BND, prostatic obstruction, PRES, and urethral stricture, are presented in Table 2. Among patients with CNS disorders, 17 (45.9%) had BND and 18 (48.6%) had a tight external sphincter. Patients with PRES had a tight ES during voiding, whereas those with idiopathic DU were significantly older in age and did not have BOO in VUDS.

**Table 2:** Clinical diagnosis of the etiology of detrusor underactivity and videourodynamic parameters in male patients.

	CNS (n = 37)	BOO (n = 122)	PRES/DV (n = 49)	Idiopathic (n = 82)	PNS (n = 8)	Normal (n = 30)
Age	63.8 ± 13.7	72.4 ± 12.8	67.9 ± 15.1	75.6 ± 12.0	59.5 ± 9.9	59.9 ± 5.9
FSF/CBC %	57.0 ± 18.0	61.5 ± 23.6	56.4 ± 17.3	59.8 ± 20.5	58.3 ± 28.7	46.1 ± 15.4
FS/CBC %	84.3 ± 20.2	87.6 ± 15.2	83.1 ± 14.4	87.5 ± 15.0	88.1 ± 13.3	75.7 ± 13.5
US/CBC %	98.7 ± 5.85	98.9 ± 5.31	98.1 ± 6.16	99.5 ± 2.60	100 ± 0.0	94.5 ± 12.1
CBC (ml)	384 ± 150	427 ± 184	353 ± 151	331 ± 203	432 ± 227	502 ± 108
PVR/CBC %	111 ± 44.3	106 ± 43.5	114 ± 116*	82.5 ± 122	160 ± 93.6	4.0 ± 5.5
Pves (cmH_2_O)	26.8 ± 22.9	33.1 ± 35.0	38.6 ± 31.5	34.6 ± 28.6	41.1 ± 36.4	50.4 ± 30.5
Pdet (cmH_2_O)	6.97 ± 7.73	10.0 ± 12.0	8.65 ± .65	12.5 ± 10.8	3.13 ± .22	32.6 ± 12.6
Qmax (ml/s)	1.0 ± 2.63	1.52 ± 2.78	2.06 ± .30	4.22 ± 3.96	0.25 ± 0.46	20.1 ± 3.1
Volume (ml)	24.3 ± 70.7	41.5 ± 91.1	52.1 ± 101	110 ± 114	16.4 ± 0.6	485 ± 103
PVR (ml)	359 ± 154	383 ± 197	301 ± 159	221 ± 227	415 ± 240	17.3 ± 24.8
Compliance	72.2 ± 93.4	73.2 ± 107	61.2 ± 87.0	62.8 ± 81.8	65.0 ± 97.6	68.2 ± 57.3
BCI	12.0 ± 18.2	17.6 ± 21.3	19.0 ± 19.6	33.6 ± 24.7	4.38 ± 4.87	133 ± 22.8
VE (%)	5.74 ± 14.8	10.9 ± 21.3	14.5 ± 24.5	41.6 ± 35.3	5.65 ± 10.2	96.7 ± 4.56
DO	5 (13.5%)	24 (19.7%)	5 (10.2%)	28 (34.1%)	1 (12.5%)	1 (16.7%)
Tight BN	17 (45.9%)	94 (77.0%)	1 (2.0%)	1 (1.2%)	4 (50%)	0 (0%)
Tight PU	3 (8.1%)	32 (26.2%)	1 (2.0%)	0 (0%)	0 (0%)	0 (0%)
Tight ES	18 (48.6%)	23 (18.9%)	44 (89.8%)	0 (0%)	0 (0%)	0 (0%)
ISD	0 (0%)	0 (0%)	2 (4.1%)	17 (20.7%)	0 (0%)	0 (0%)

*CNS* central nervous system disorders, *BOO* bladder outlet obstruction, *PRES* poor relaxation of external sphincter, *PNS* peripheral nervous system disorders, *FSF* first sensation of filling, *CBC* cystometric bladder capacity, *FS* full sensation, *US* urge sensation, *PVR* post-void residual, *Pves* intravesical pressure, *Pdet* detrusor pressure, *Qmax* maximum flow rate, *BCI* bladder contractility index, *VE* voiding efficiency, *DO* detrusor overactivity, *BN* bladder neck, *PU* prostatic urethra, *ES* external sphincter, *ISD* intrinsic sphincter deficiency.

According to the VUDS findings, conservative treatment (indwelling catheter, CIC, cystostomy voiding training), bladder outlet surgeries (TUI-BN, TUI-P, TURP, optic urethrotomy), urethral sphincter BoNT-A injection, or medical treatment were given to individual DU patients. The treatment outcomes and relationship with VUDS findings are presented in Table 3. Overall, 68.4% of the patients received bladder outlet surgery, 59.1% received urethral BoNT-A injection, and 57.6% who received medical treatment had satisfactory outcomes (with VE > 66.7%); however, 72.7% of the patients with conservative treatment could not regain spontaneous urination during the follow-up period (*p* = 0.000).

**Table 3:** Treatment outcome of different managements in patients with detrusor underactivity.

	N =	Grade 0	Grade 1	Grade 2	Grade 3
Conservative, CIC and indwelling catheter	44	32 (72.7%)	4 (9.1%)	7 (15.9%)	1 (2.3%)
Medical treatment	99	11 (11.1%)	31 (31.3%)	38 (38.4%)	19 (19.2%)
Bladder outlet surgeries	133	14 (10.5%)	28 (21.1%)	31 (23.3%)	60 (45.1%)
Urethral sphincter BoNT-A injection	22	5 (22.7%)	4 (18.2%)	11 (50%)	2 (9.1%)
Total	298	62 (20.8%)	67 (22.5%)	87 (29.2%)	82 (27.5%)

Grade 0: VE < 33.3%, Grade 1: VE 33.3–66.7%, Grade 2: VE 66.7–90%, Grade 3: VE > 90%; Grade 0 vs 1–3, *p* = 0.000; Grade 0 + 1 vs 2 + 3, *p* = 0.000.

*CIC* clean intermittent catheterization, *BoNT-A* botulinum toxin A.

Compared with conservative treatment (by CIC or medical treatment), active treatment for patients with VUDS-proven tight bladder neck, narrow prostatic urethra, and PRES is usually effective. The improvement rates of patients with marked (GRA = 3) and moderate improvement (GRA = 2) after management, those who had NDC (81.8%), and those who had LDC (69.2%) were better than the rates of the patients with DA (41.3%), whereas the improvement rates of patients with IBS (FSF/CBC < 30%) and of those with normal bladder sensation (FSF/CBC 30%–50%) (78.1%) was better than the rates of patients with reduced bladder sensation (48.7%) and the rates of patients with absent bladder sensation (17.9%) (Table 4). The baseline PVR volume did not affect the treatment outcome. The BOO appearance during VUDS usually indicated a high urethral resistance. Among 164 patients with a tight bladder outlet, 62.8% had an improvement outcome (grade 2 or 3), 130 patients received bladder outlet surgeries and 88 (67.7%) had an improved outcome.

**Table 4:** Relationship of videourodynamic parameters and treatment outcome.

	N	Grade 0	Grade 1	Grade 2	Grade 3	p value
NDC (Pdet ≥ 30 cmH_2_O)	22	1 (4.5%)	3 (13.6%)	7 (31.8%)	11 (50%)	0.000
LDC (Pdet < 30 cmH2O)	133	12 (9.0%)	29 (21.8%)	48 (36.1%)	44 (33.1%)	
DA (Pdet ≤ 5 cmH2O)	143	49 (34.3%)	35 (24.5%)	32 (22.4%)	27 (18.9%)	
FSF/CBC = 100%	28	16 (57.1%)	7 (25%)	4 (14.3%)	1 (3.6%)	0.000
≥ 50%	160	35 (21.9%)	47 (29.4%)	45 (28.1%)	33 (20.6%)	
< 50%	110	11 (10%)	13 (11.8%)	38 (34.5%)	48 (43.6%)	
PVR < 250 ml	117	21 (17.9%)	22 (18.8%)	28 (23.9%)	31 (26.5%)	0.631
PVR ≥ 250, < 500 ml	92	18 (19.6%)	23 (25%)	35 (38.0%)	25(27.2%)	0.748
PVR ≥ 500 ml	89	19 (21.3%)	22 (24.7%)	22 (24.7%)	26 (29.2%)	
Presence of BOO	164	19 (11.6%)	41 (25%)	43 (26.2%)	61 (36.6%)	0.000
Tight ES/DV	43	14 (32.6%)	10 (23.3%)	15 (34.9%)	4 (9.3%)	0.028
No definite BOO	91	29 (31.9%)	16 (176%)	29 (31.9%)	17 (18.7%)	

Grade 0: VE < 33.3%, Grade 1: VE 33.3–66.7%, Grade 2: VE 66.7–90%, Grade 3: VE > 90%

*BOO* bladder outlet obstruction, *ES* external sphincter, *PVR* post-void residual, *NDC* normal detrusor contractility, *LDC* low detrusor contractility, *DA* detrusor acontractile, *FSF* first sensation of filling, *CBC* cystometric bladder capacity, *DV* dysfunctional voiding.

Table 5 shows the combination of the detrusor contractility (DA, LDC, and NDC) and bladder sensation (absent and reduced, normal, and increased) and compared the treatment outcomes (with grade 2 and grade 3 improvement) in different DU subgroups. Patients with normal or increased bladder sensation had the best treatment outcome regardless of the detrusor contractility (DA 63.3%, LDC 92.3%, and NDC 77.8%). Patients with absent or reduced bladder sensation and DA (29.8%) or LDC (54.3%) had a worse treatment outcomes. Among the 24 patients with DA and absent bladder sensation, a grade 2 or 3 improvement was found only in three (12.5%) patients.

**Table 5:** Treatment outcome of DU patients according to different combinations of detrusor contractility and bladder sensation.

	N	Grade 0	Grade 1	Grade 2	Grade 3
DA/reduced BS*	94	39 (41.5%)	27 (28.7%)	17 (18.1%)	11 (11.7%)
DA/normal BS^#^	49	10 (20.4%)	8 (16.3%)	15 (30.6%)	16 (32.7%)
LDC/reduced BS	81	11 (13.6%)	26 (32.1%)	27 (33.3%)	17 (21.0%)
LDC/normal BS	52	1 (1.9%)	3 (5.8%)	21 (40.4%)	27 (51.9%)
NDC/reduced BS	13	1 (7.7%)	1 (7.7%)	5 (38.5%)	6 (46.2%)
NDC/normal BS	9	0 (0%)	2 (22.2%)	2 (22.2%)	5 (55.6%)

Grade 0: VE < 33.3%, Grade 1: VE 33.3–66.7%, Grade 2: VE 66.7–90%, Grade 3: VE > 90%. Reduced BS* contains reduced and absent bladder sensation, normal BS^#^ contains normal and increased bladder sensation. *DA* detrusor acontractile, *LDC* low detrusor contractility, *NDC* normal detrusor contractility. Grade 0 vs 1–3, P = 0.000; Grade 0 + 1 vs 2 + 3, *p* = 0.000.

When we compared the treatment outcomes among different DU subgroups by VUDS findings and the treatment modalities, we found that BOO surgeries were effective in resuming effective voiding in most DU patients except those with DA (58.8%), absent bladder sensation (26.7%), and reduced bladder sensation (58.5%). Medical treatment was also effective in DU patients with NDC (66.7%) and LDC (72.7%) but not in DA (32.4%) or patients with absent bladder sensation (33.3%). For patients receiving conservative treatment with CIC, an indwelling catheter usually did not enable them to resume spontaneous voiding, but in some patients with LDC (40%) and normal bladder sensation (35.7%), an indwelling catheter might be effective. A urethral BoNT-A injection was effective in DU patients with NDC (100%) or LDC (75%) with normal bladder sensation (100%) and without BOO (100%) in VUDS (Table 6).

**Table 6:** Distribution of improved treatment outcome (with grades 2 and 3 improvement) among different VUDS subgroups and treatment modalities.

	N	Conservative treatment	Medical treatment	BOO surgery	BoNT-A injection
Total	298	8/44 (18.2%)	57/99 (57.6%)	91/133 (68.4%)	13/22 (59.1%)
NDC	22	0/1 (0%)	6/9 (66.7%)	11/11 (100%)	1/1 (100%)
LDC	133	6/15 (40%)	40/55 (72.7%)	40/54 (74.1%)	6/8 (75%)
DA	143	2/28 (7.1%)	11/34 (32.4%)	40/68 (58.8%)	6/13 (46.2%)
**FSF/CBC = 100%**	28	1/10 (10%)	1/3 (33.3%)	4/15 (26.7%)	–
≥ 50%	160	2/20 (10%)	33/61 (54.1%)	38/65 (58.5%)	5/14 (35.7%)
< 50%	110	5/14 (35.7%)	24/34 (70.6%)	49/53 (92.5%)	8/8 (100%)
PVR < 250 ml	108	1/11 (9.1%)	34/57 (59.6%)	23/36 (63.9%)	3/4 (75%)
PVR ≥ 250 < 500 ml	101	4/15 (26.7%)	16/29 (55.2%)	34/44 (77.3%)	6/12 (50%) 4/6 (66.7%)
PVR ≥ 500 ml	89	3/18 (16.7%)	6/12 (50%)	34/53 (64.2%)	
BND, BPO, US	164	2/8 (25%)	12/26 (46.2%)	88/130 (67.7%)	–
Tight ES, DV	43	0/7 (0%)	7/15 (46.7%)	2/2 (100%)	10/19 (52.6%)
No definite BOO	91	6/29 (20.7%)	36/56 (64.3%)	1/1 (100%)	3/3 (100%)

*BOO* bladder outlet obstruction, *PVR* post-void residual, *NDC* normal detrusor contractility, *LDC* low detrusor contractility, *DA* detrusor acontractile, *FSF* first sensation of filling, *CBC* cystometric bladder capacity, *BND* bladder neck dysfunction, *BPO* benign prostatic obstruction, *US* urethral stricture, *ES* external sphincter, *DV* dysfunctional voiding, *BoNT-A* botulinum toxin A.

## Discussion

DU has many different etiologies, including psychogenic, neurogenic, myogenic, and reduced detrusor contractility after increasing muscle work against anatomical or functional BOO^2,8,9,11^. Lack of sensory input from the bladder and facilitative activity from the CNS also contribute to DU^12,16,17^. Among patients with DU, some will recover after conservative treatment with an indwelling catheter, CIC, or an alpha-blocker. Since there is no suitable medical treatment to enhance bladder contractility, the effective treatment depends on relief of bladder outlet resistance and can be TUI-BN, TURP, or urethral sphincter BoNT-A injection. However, patients with DU usually have reduced bladder sensation, LDC, and large PVR. It is difficult to identify DU patients who are likely to recover after a specific urological procedure. This study revealed that normal bladder sensation, presence of adequate detrusor contractility, and bladder outlet condition during VUDS could be effective treatment strategies for DU patients.

The study results also revealed that most of the DU patients had reduced bladder sensation, which is closely associated with low detrusor contractility. After active treatment, 68.4% of the patients benefited from BOO surgery, 59.1% from urethral Botox injection, and 57.6% from medical treatment, but only 18.2% benefited from conservative treatment. The patients with detrusor contractility, either LDC or NDC, and normal or IBS had greater improvement after any kind of urological management, suggesting that residual detrusor energy and presence of afferent innervation provide adequate recoverability of detrusor contractility.

Among different urological managements of DU, BOO surgery provides the highest improvement rate (68.4%). Although chronic BOO could result in detrusor decompensation, it is still difficult to differentiate BOO in patients with DU, especially in those with absent bladder sensation and DA. Nevertheless, this study revealed that most male DU patients benefited from BOO surgery as long as they still had a LDC or NDC and presence of bladder sensation. Even in patients with DA, BOO surgery still provided improved treatment outcome for 58.8% of the DU patients. Recent studies have shown that patients with DU with or without BOO can still benefit from transurethral prostatic surgeries and have improvement of LUTS after surgery^18,19^. However, patients with DA might need CIC after prostatic surgery^19^, and patients with DU are more likely to have a lower quality of life and failure to voiding in the immediate postoperative period^20^.

Our previous study had shown that patients with DU and received transurethral prostatic surgery had very good improvement of voiding, especially in the patients who had a greater bladder compliance and a higher Pdet^21^. In another study, we also found that the recovery of detrusor contractility can be achieved after prostatic surgery if patients with DU and small prostate had a higher voiding Pdet and Qmax at baseline^22^. About 60% of DU patients could have a positive surgical outcome after prostatic surgery. Among these patients, 72.4% of patients had detrusor function recovery within the first month. The VUDS at baseline could predict surgical outcome and identify suitable DU patients for the prostatic surgery.

A normal micturition needs intact bladder sensation which transmits the afferent stimuli from stretch receptors of the bladder wall and excites the efferent pathway to contract the detrusor muscles. The normal sensory activation of the micturition reflex is necessary for normal detrusor contractility during micturition^23^. Some patients with clinical DU might be due to absent or diminished bladder sensation, resulting in inability to initiation or early termination of the completeness of voiding process^12^. From the electromyographic study of patients with DU and chronic urinary retention, we found the causes of DU included bladder dysfunction, neuropathy in sacral reflexes, pudendal nerve, and urethral sphincter innervation^24^. Bladder sensation was decreased and CBC was larger in the DU group than in the detrusor hyperactivity and inadequate contractility, hypersensitive bladder, and DO groups. Bladder sensation was also negatively associated with detrusor contractility. BCI and VE were lower in the DU patient groups^4^. This study found that patients with absent or reduced bladder sensation had limited treatment outcomes. In contrast, patients with normal or increased bladder sensation could benefit from BOO surgery, urethral BoNT-A injection, or medical treatment. Patients with reduced bladder sensation might not perceive bladder fullness and mediate detrusor contractility, so they might continue to have difficult urination or a large PVR and a low VE after treatment.

Patients with a severe cortical degenerative disease may lack bladder perception and be unable to initiate voiding. In this study we noted DA patients might have more reduced bladder first sensation and very LDC. In these patients, treatment outcome was also less improved than in patients with increased or normal bladder sensation. Patients without normal bladder sensation usually cannot adequately empty their bladders by detrusor contraction or abdominal straining even though the urethral resistance has been reduced after BOO surgery^25,26^. Clinically, the voiding training result was better in patients without normal bladder sensation than in DU patients with absent or reduced bladder sensation and could usually be expected to be acceptable once the patients with chronic urinary retention regained normal bladder fullness sensation.

The causes of Idiopathic underactive bladder in the old aged male patients include DU and PRES based on the video urodynamic study. Most patients with DU are associated with aging, diminished or absent bladder sensation, lower detrusor contractility, and comorbidities such as diabetes, chronic kidney disease, and congestive heart failure^3^. Interestingly, we also noticed that patients with detrusor acontractile and chronic urinary retention could regain their detrusor contractility and voiding ability after medical or surgical treatment. A total of 43.9% of patients with DA could have detrusor function recovery after treatment, a bladder compliance of less than 80 ml/cmH_2_O could predict the recovery^27^. These patients might have residual detrusor contractility and had urodynamically higher Pdet and lower bladder compliance, suggesting these patients did not completely lose their detrusor function and had a better chance to regain effective detrusor contractility and adequate voiding efficiency^21^. In this study, DU patients with LDC and NDC had better treatment outcomes regardless of the types of urological treatment, which further implied the predictive factor of residual detrusor contractility in the decompensated bladder to regain effective contractility of voiding.

In male patients with DU, the differential diagnosis between bladder out obstruction and true detrusor failure is difficult because patients usually cannot urinate with adequate detrusor pressure. However, from VUDS we can still observe a tight bladder neck or prostatic urethra during voiding attempts. The results of this study showed that if a VUDS found a tight BN, prostatic urethra, or urethra, BOO surgery could resume spontaneous voiding in 67.7% of DU patients, which was better than in patients receiving conservative treatment or medical therapy. Ablation and resection of the bladder neck and prostate urethra might decrease the sympathetic activity that usually inhibits bladder contractility, resulting in detrusor function recovery^28^.

If DU patients were found to have a narrow urethral sphincter during voiding in a VUDS, urethral sphincter BoNT-A was recommended and a 52.6% improvement rate still could be obtained. A urethral sphincter injection of BoNT-A has been reported to be effective in 60% of patients with DU^29^. A tight bladder neck during voiding by either detrusor contraction or abdominal pressure is an unfavorable prognostic factor for successful BoNT-A treatment^30^. Urine cannot be adequately expelled by abdominal straining when the bladder neck is tight, so a TUI-BN or TUI-P prior to BoNT-A injection might be necessary to achieve a satisfactory treatment outcome^30,31^.

A previous study showed that patients with DU and normal bladder sensation combined with a poor relaxation or hyperactive urethral sphincter were significantly more likely to recover normal detrusor function after urethral sphincter BoNT-A injection^32^. In this study, we also observed that more DU patients with NDC or LDC benefited from BoNT-A injection than the number of patients with DA. Careful VUDS interpretation of the bladder neck and prostatic urethral opening as well as bladder sensation enables urologists to select appropriate candidates for urethral sphincter Botox treatment.

In this study, we found that DU patients who received conservative treatment had the worst treatment outcomes. It is likely that these patients had more medical comorbidities and poor general condition. In patients who had DU due to BOO, PRES, or idiopathic detrusor failure, medical treatment still can be effective in a certain percentage of patients. Although the urodynamic study revealed no definite bladder neck or prostatic urethra narrowing, alpha-blocker still can relax the urethra, and might provide a chance to improve VE. The concept is similar to that unobstructed patients with DU can still benefit from TURP^18^. However, patients with absent bladder sensation and very LDC still might not have good treatment outcomes after conservative or medical treatment. Appropriate bladder management either by CIC or cystostomy training is also important in the initial stage of management to avoid bladder overdistention and resume normal blood perfusion. DU might be a result of chronic bladder overdistention and ischemia. It is mandatory to avoid bladder injury due to repeat overdistention during voiding training.

## Conclusion

VUDS provides an understanding of bladder sensation, detrusor contractility and bladder outlet condition during voiding in patients with DU. The VUDS finding can also provide guidance for appropriate urological management. Patients with very low detrusor contractility and absent bladder sensation usually had less favorable treatment outcomes for conservative treatment, BOO surgery, or urethral sphincter BoNT-A injection. Among all managements, BOO surgery provided the best treatment outcome.
